# The effect of educational intervention based on health belief model and social support on testicular self-examination in sample of Iranian men

**DOI:** 10.1186/s12885-021-08411-5

**Published:** 2021-06-10

**Authors:** Ali Khani Jeihooni, Hanieh Jormand, Mehdi Ansari, Pooyan Afzali Harsini, Tayebeh Rakhshani

**Affiliations:** 1 Departement of Public Health, School of Health, Shiraz University of Medical Sciences, 7153675541 Shiraz, Iran; 2 Departement of Health Education and Promotion, School of Health, Hamadan University of Medical Sciences, Hamadan, Iran; 3 Departement of Public Health, School of Health, Fasa University of Medical Sciences, Fasa, Iran; 4 Department of Public Health, School of Health, Kermanshah University of Medical Sciences, Kermanshah, Iran

**Keywords:** Health belief model, Fasa city, Social support, Testicular cancer, Testicular self-examination

## Abstract

**Background:**

Testicular Self-Examination (TSE) causes earlier diagnosis of Testicular cancer (TC). Hence, all men aged between15 to 35 years should perform TSE every month. This study aims to survey the effect of educational intervention based on health belief model and social support on testicular self-examination in men aged between 15 to 35 years of Fasa City, Fars province, Iran.

**Methods:**

In this quasi-experimental study, 200 men (100 in the experimental group and 100 in the control group) in Fasa City, Fars, Iran, were conducted from June 2018 to August 2019. The educational intervention for the experimental group consisted of six training sessions (testicular cancer, its prevalence and types, its risk factors, symptoms, infected areas, diagnosis, side-effects and its severity, understanding about testicular self-examination and its importance, benefits, and barriers of self-examination and correct way of doing TSE were discussed, role of social support). A questionnaire consisting of demographic information, knowledge, HBM construct, and social support was used to measure testicular self-examination before, 3 months after the intervention, and 6 months later. Data were analyzed using SPSS-22 via chi-squared, independent samples t-test, Mann-Whitney, and repeated measures ANOVA at a significance level of 0.5.

**Results:**

The mean age of the men was 27.26 ± 3.16 years in the experimental group and 27.39 ± 3.12 years in the control group. Three months after the intervention and 6 months after the intervention, the experimental group showed a significant increase in knowledge, perceived susceptibility, perceived severity, perceived benefits, self-efficacy, cues to action, social support, and testicular self-examination performance compared to the control group.

**Conclusion:**

This study showed the effectiveness of the intervention based on the HBM constructs and social support in the adoption of testicular self-examination in 3 and 6 months post-intervention in men aged between 15 to 35 years. Hence, these models can act as a framework for designing and implementing educational interventions for testicular self-examination.

## Background

Testicular cancer (TC) is the most common neoplasm among young men [1, 2]. Although accounting for only 1 to 2% of male cancers, testicular cancer is the most commonly diagnosed cancer in men aged between 15 and 35 years in the United States and European populations [3].

Testicular cancer is generally represented as a nodal or inflammation without pain in one of the testicles which may be detected by the patient or his sexual partner. Sometimes a person who had testicular atrophy or largeness of the testicle might be infected by testicular cancer. Almost 30 to 40% of patients complain about unclear pain and heaviness in the lower abdomen, perineal area, or scrotum. In 10% of patients, testicular cancer symptoms are very severe. In 10% of patients, the clinical signs of TC depend on the metastatic phase of illness and the infected areas. Based on the infected areas, signs are different:

- A cervical mass (Metastasis of the lymph nodes of upper Corsets).
- Coughing or shortness of breath (Metastasis of lungs).
- Anorexia, nausea and vomiting, bleeding in the digestive system (Metastasis of posterior duodenum).
- Backache (a bulky retroperitoneal disease that affects Psoas major and the root of the lumbar nerves).
- Skeletal pain (osseous metastasis).
- Symptoms in central or peripheral nerve system (brain involvement, spinal cord, and peripheral nerves).
- One-side or two-side inflammation of lower extremity (Thrombosis or blockage of cavil or iliac arteries) [4–7].

The most prevalent symptom of TC is the inflammation of the testicle without any pain. The most important factor is the stage of cancer when it is diagnosed [8–10].

Unfortunately, most men have a delay in referring to a doctor which is mostly due to the lack of knowledge about symptoms of TC or ignoring them and it is mostly based on this fact that the signs of TC are generally subsidiary, including a nodal or wounding, heaviness in testicle sack, feeling unwell and pain (which are less common). Therefore, the lack of initial diagnosis is very possible. This delay causes 50 to 88% of men suffering from TC to enter the metastatic phase in which the side-effects and death rate are highly significant [11–14].

Theoretically, a regular touch of testicles by the individual (self-examination) or by a specialist (clinical observation) helps the diagnosis of TC before appearing TC symptoms. In the past, doctors and health care providers encouraged adult men to learn and perform testicular self-examination (TSE) [15, 16].

TSE is in this way that testicle places between pollex, point, and middle fingers. Every sign including stiffness, mass, or inflammation with no pain should be considered seriously and needs lateral observations. TSE should be performed periodically (once a month for example) [17, 18].

A study reported that 89% of adult men had never performed TSE and only 4% of them knew that young men should perform TSE every month [19]. Literature shows that the lesion can be easily detected in early stages through TSE and can be effectively managed [18], but health care providers seldom teach TSE, thus it causes potential missed opportunities for early detection. Therefore, this issue indicates that the school environment might provide the best setting to teach TSE [19, 20]. Ramim et al. [21] reported that only 7.9% of Iranian students did self-examination. Wardle et al. [22] reported that only 3% of students did self-examination every month.

TC is an unavoidable disease, however, regular examination of testicles causes earlier diagnosis of TC. Hence, all men aged between15 to 35 years should perform TSE every month [9]. According to the importance of TC, providing programs for promoting TSE behaviors becomes more demanded.

### Theoretical frameworks

For this reason, educating and increasing the knowledge of people are the fundamental programs for promoting TSE behavior [23]. The worth of educational programs depends on their efficiency and their efficiency mostly depends on the appropriate use of theories and health education models. In other words, selecting a pattern for health education is the first step in the planning process of every health education program. A proper pattern keeps the program in its correct direction. One of the effective models in health education and promotion is the health belief model and its constructs are perceived susceptibility, perceived severity, perceived benefits, perceived barriers, cues to action, and self-efficacy [24]. In this model, the perceived susceptibility is the attitude of men aged between 15 to 35 years about this point that how much they think they might be infected by TC. Also, the perceived severity estimates the attitude of men about the severity and side-effects of TC, and the sum of these two factors is the perceived threat of men about TC. The perceived threat along with perceived benefits and barriers is the analysis of the advantages of TSE behaviors and the analysis of barriers for performing TSE along with the perceived power of men for taking TSE behaviors. Also, cues to action or the internal or external motivations (such as friends, relatives, doctors, or fear from TC side-effects) lead people to perform TSE [13, 25].

The health belief model is mostly used to collect data on individual behavior variables. However, other factors can lead to behavior [26, 27]. When the educational program for modifying preventive behaviors is flexible and proportional to individuals’ characteristics, it becomes successful. For compensating for the faults of the health belief model, the social support construct from social cognitive theory was employed in this investigation. The studies indicated that social support has a positive effect on different aspects of preventive activities from skin cancer [28]. Social support is “the facilities in which others provide for an individual”. Also, this definition is considered as a factor that makes a person believe that he/she is respected and loved by others, he/she is a worthy person and belongs to a social community with corresponding relationships and obligations. Social supports can be investigated through the evaluation of other’s behavior and it is provided by various sources such as specialists, families, and friends [29].

There are numerous studies about TSE performance by using the health belief model and social supports such as the studies of Vadaparampil et al. [30], McClenahan et al. [13], and Asgharpour et al. [31]. According to the importance of TSE, it can be concluded that this issue plays an important role in TC prevention and it also prevents the initial and treatable stages of TC from developed and uncontrollable stages. Since the interventional study based on the health belief model about TC has not been carried out in Iran, the purpose of this study is to determine the effect of educational intervention based on the health belief model and social support on TSE in men aged between 15 to 35 years under the coverage of health centers of Fasa city, Fars province, Iran.

## Methods

### Design and sample

**Fig. 1:**
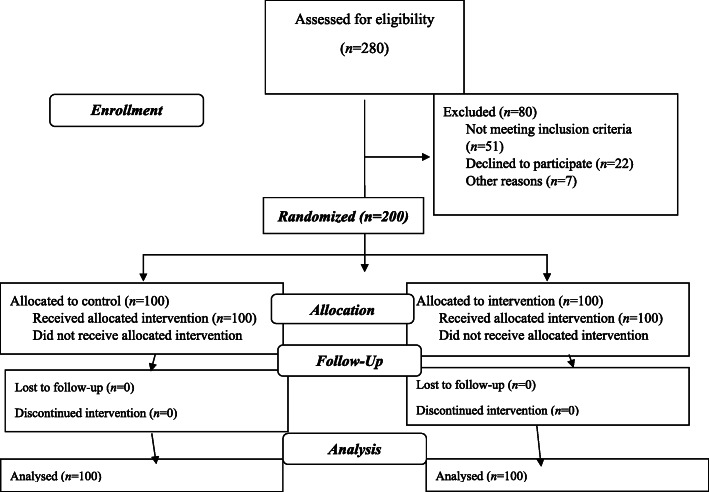
Flow chart of study

This research is a quasi-experimental study conducted from June 2018 to August 2019. The subjects of this study were 200 men aged between 15 to 35 years who were under the coverage of Fasa Health Centers. Two out of six Health Centers were randomly selected (one center for the control group and the other one for the experimental group). In each Health Center, 100 subjects were selected based on the number of their family files recorded in Health Centers (100 patients in the experimental group and 100 in the control group). Figure 1 was presented in a flow diagram.

The inclusion criteria of the present research were men aged between 15 to 35 years who were not infected by TC and residents in Fasa city. The exclusion criteria were the men were suffering from other chronic systemic illnesses urology, the lack of interest of subjects for participating in the study and being absent in more than two sessions. This study was approved by the Ethics Committee of Fasa University of Medical Sciences. Informed consent was taken from all the participants. Although was emphasized that the participants were given the right to leave the study at any time if they wished to leave it.

Using the data of previous studies [31], the sample volume was estimated by using the difference ratio formula of 95%, power 80%, the average score of the perceived benefits of TSE before educational intervention 10.68 ± 2.8 and after the intervention 11.74 ± 2.41 and due to the possibility of samples drop, the number of samples for each group was considered 100 subjects.

### Study instrument and measures

The data collection tools were a designed and validated questionnaire by McClenahan et al. [13], Avci and Altinel [25], and Akar and Bebis [32]. The questionnaire was anonymous and coded arranged in 5 sections. The first section included 7 demographic questions about age, marital status, educational level, smoking, alcohol consumption, performing TSE, and the history of testicular cancer in the family. The second section included 10 items for evaluating knowledge in which the “yes” answer had 1 score and the “no” or “no idea” answer had a zero score. The third section was related to the evaluation of health belief model constructs. In this section, 5 items were about the perceived susceptibility, (For example, “The possibility that I have testicular cancer is very high”), 10 items for perceived severity, (For example, “If I have testicular cancer, my whole life will change.”), 6 items for perceived benefits, (For example, “If I do self-examination every month, I will be less likely to die because of testicular cancer.”), 9 items for perceived barriers, (For example, “Testicular self-examination is painful.”), 5 items for cues to action, (For example, “Doctor’s recommendations prompted me to do testicular self-examination.”), 6 items for motivation, (For example, “I have a well-balanced diet.”) and 8 items for self-efficacy. (For example, “I can do TSE correctly.”). Also, the fourth section included 5 items for evaluating the social support constructs. (For example, “my family who is very important to me would approve my TSE in the following month.”).

Questions about health belief model constructs and social support were arranged based on a five-point Likert scale, so that, “completely agree” had the score of 5, “agree” had the score of 4, “no idea” had the score of 3, “disagree” had the score of 2 and “completely disagree” had the score of 1. The range of scores for perceived susceptibility, motivation, social support, and cues to action was from 5 to 25, for self-efficacy was from 6 to 30, for perceived severity was from 10 to 50, for perceived benefits was from 6 to 30 and for perceived barriers was from 9 to 45.

The fifth section included 5 items about testicular self-examination as “yes or no” questions which were answered through self-reporting (For example, “I perform testicular self-examination at least once a month.”) and the range of scores was from 0 to 5. All scores were reported in percentage.

To evaluate the validity of the questionnaire items, item effect size higher than 0.15 and content validity ratio above 0.78 were considered and based on the exploratory factor analysis. In order to determine the face validity of the tool, a list of arranged items was proposed by 25 men aged between15 to 35 years with similar demographic, economic, and social characteristics. To determine the content validity, the ideas of 12 specialists (out of the research team) in health education and promotion (10 people), urologist (1 person), and biostatic (1 person) were employed. Based on Lawshe’s table, items with a CVR value higher than 0.56 for 12 people were considered acceptable and were retained for subsequent analysis. In the current investigation and most of the items, the calculated values were higher than 0.70. The total reliability of the research tool with the calculation of Cronbach’s alpha was 0.87. the perceived susceptibility was 0.86, perceived severity was 0.80, perceived benefits was 0.79, perceived barriers was 0.83, cues to action was 0.80, self-efficacy was 0.78 and social support was 0.79. The questionnaire was filled out before the educational intervention and 3 and 6 months after the intervention by both of the experimental and control groups. All subjects participated from the beginning to the end of the investigation.

### Developed educational intervention

The educational intervention for the experimental group included 6 educational sessions (50–55 min) by giving representation, group discussions, asking and answering questions, and also using posters and educational pamphlets, films, and power points. It is essential to mention the number of sessions was specified based on a literature review in this field [32, 33]. Moreover, the first session’s focused knowledge about testicular self-examination, the second session’s focused on perceived benefits and perceived barriers to testicular self-examination, the third session emphasized perceived benefits and perceived barriers to testicular self-examination, the fourth session stressed cues to action and motivation to testicular self-examination, the last session highlighted self-efficacy and social support. The educational program was performed by a Ph.D. in health education and promotion and one urologist. In these sessions, testicular cancer, its prevalence and types, its risk factors, symptoms, infected areas, diagnosis, side-effects and its severity, understanding about testicular self-examination and its importance, benefits, and barriers of self-examination, and correct way of doing TSE were discussed.

In one of the educational sessions, a 58 years old man suffering from TC was invited to talk about TC, its risk factors, symptoms, and side effects and also, the importance of TSE. One educational session was also held with the presence of one of the family members and health specialists and the role of social supports for taking and simplifying TSE performance was emphasized.

Participants of the intervention group were divided into groups with 20 members for reinforcement social support constructs (helper group and friend group) and the educational program was performed for 5 groups with 20 members (100 subjects of intervention group) in the salon of the health center once a week. This division provided both sharing information in a friendly atmosphere and depending on the issue, persons feel more comfortable together. At the end of educational sessions, an educational booklet was given to the subjects. It is essential to be mentioned, the choice to join a friend or helper group was left to the individual and was entirely optional. Also, every week an educational and motivational message about the importance of TSE was sent to the subjects and a telegram group was provided for information exchange. In two and four months after the educational intervention, one follow-up session was held for reviewing the contents and activities of subjects. Moreover, at the end of the study, one educational session was held for the control group and an educational booklet was given to them.

For ethical considerations, in addition to the license from the ethical committee of Fasa University of medical science and health center of Fasa city, the studied subjects were informed about the aims, importance, and essentiality of this research, and a written content letter was filled out by subjects and they were ensured that their information would remain confidential.

### Data analysis

By using SPSS 22 software, Chi-square static tests, Independent t-test, Mann-Whitney, and Repeated Measurement ANOVA, the obtained data were analyzed and the significant level of 0.05 was considered. The Mann–Whiney test and the Independent t-test, were used to compare frequency distribution of experimental and control groups characteristics. The Kolmogorov-Smirnov test showed that Education variable was not normally distributed, the Mann–Whiney U test was used to compare of frequency distribution of experimental and control groups characteristics.

## Results

In this study, 200 men aged between 15 to 35 years under the coverage of health centers in Fasa city (100 subjects for the experimental group and 100 subjects for the control group) were investigated. The average age of studied subjects of the experimental group was 27.26 ± 3.16 years and the control group was 27.39 ± 3.12 years and according to the independent t-test, the experimental and control group had no significant differences (*p* = 0.109). Almost 5% of the experimental group and 3% of the control performed the testicular self-experiment before. Other demographic information of the subjects of the two groups showed no significant differences (Table 1).

**Table 1:** The comparison of frequency distribution of experimental and control groups characteristics

variables		experimental group (*n* = 100)		Control group (n = 100)		*P*-Value
Marital status	single	number	percent	number	percent	0.342^*a^
		52	52	50	50	
	married	48	48	50	50	
Educational level	elementary	6	6	4	4	0.267^*b^
	Guidance school	14	14	13	13	
	High school	46	46	48	48	
	university	34	34	35	35	
History of smoking	yes	39	39	33	33	0.288^*a^
	no	61	61	67	67	
History of alcohol consumption	yes	24	24	27	27	0.325^*a^
	no	76	76	73	73	
History of TC in family	yes	5	5	2	2	0.451^*a^
	no	95	95	98	98	
History of TSE before	yes	5	5	3	3	0.462^*a^
	no	95	95	97	97	

^a^ *p*-Values based on the independent samples t-test

^b^p-Values based on the Mann–Whitney U-test

*The level of significance was set at *P* < .05

The obtained results revealed that, before the educational intervention, there were no significant differences between experimental and control groups in knowledge, perceived susceptibility, perceived severity, perceived benefits, cues to action, perceived motivation, self-efficacy, social supports, and testicular self-examination, however, 3 and 6 months after the intervention, in comparison with the control group, the experimental group indicated significant enhancement in each of the mentioned constructs, except in perceived barriers, and about the perceived barriers, the experimental group had more significant reduction than the control group (Tables 2 and 3).

**Table 2:** The comparison of average scores of health belief model construct in experimental and control groups 3 and 6 months after the intervention

variables	group	Before intervention M ± SD	3 months after intervention M ± SD	6 months after intervention M ± SD	P-Value
knowledge	experimental	26.45 ± 4.50	57.78 ±4.68	79.12 ± 4.38	0.001
	control	27.20 ± 4.31	28.42 ± 4.19	29.50 ± 4.12	0.157
	P-Value	0.246	0.001	0.001	
Perceived susceptibility	experimental	32.10 ± 4.16	64.72 ± 4.28	80.18 ± 4.40	0.001
	control	31.58 ± 4.08	32.24 ± 4.07	33.62 ± 4.15	0.178
	P-Value	0.122	0.001	0.001	
Perceived severity	experimental	30.25 ± 4.67	63.44 ± 4.68	82.51 ± 4.26	0.001
	control	31.53 ± 4.19	32.40 ± 4.27	33.58 ± 4.35	0.117
	P-Value	0.290	0.001	0.001	
Perceived benefits	experimental	22.62 ± 5.66	63.25 ± 5.50	79.42 ± 5.36	0.001
	control	23.14 ± 5.27	24.18 ± 5.20	25.68 ± 5.18	0.164
	P-Value	0.138	0.001	0.001	
Perceived barriers	experimental	80.22 ± 4.38	54.30 ± 4.52	31.23 ± 4.24	0.001
	control	81.13 ± 4.29	80.08 ± 4.28	78.95 ± 4.48	0.214
	P-value	0.235	0.001	0.001	
Cues to action	experimental	34.55 ± 4.64	64.69 ± 4.48	79.98 ± 4.64	0.001
	control	33.90 ± 4.25	34.49 ± 4.36	35.43 ± 4.22	0.108
	P-Value	0.128	0.001	0.001	
Perceived motivation	experimental	32.29 ± 4.52	61.40 ± 4.75	80.23 ± 4.36	0.001
	control	31.90 ± 4.68	32.60 ± 4.28	33.78 ± 4.31	0.147
	P-Value	0.252	0.001	0.001	
Perceived self-efficacy	experimental	25.73 ± 4.11	62.46 ± 4.51	78.75 ± 4.49	0.001
	control	26.18 ± 4.24	28.01 ± 4.03	29.48 ± 4.23	0.125
	P-Value	0.179	0.001	0.001

**Table 3:** The comparison of average scores of social supports and TSE in experimental and control groups 3 and 6 months after the intervention

variables	group	Before intervention	3 months after the intervention	6 months after the intervention	P-Value
Perceived social support	experimental	38.14 ± 4.20	68.47 ± 4.10	83.58 ± 4.33	0.001
	control	37.81 ± 4.28	38.53 ± 4.35	39.72 ± 4.29	0.148
	P-Value	0.134	0.001	0.001	
TSE performance	experimental	8.22 ± 1.35	54.27 ± 3.51	65.17 ± 3.42	0.001
	control	9.09 ± 1.32	11.40 ± 1.16	13.58 ± 1.54	0.162
	P-Value	0.155	0.001	0.001	

## Discussion

According to the importance of testicular cancer, educational intervention based on health education and promotion patterns for promoting TSE become demanded [13, 31, 32].

The results of the present study revealed that using the health belief model and social supports for educating men aged between 15 to 35 years causes the enhancement of average score of knowledge, health belief model constructs (except the perceived barriers), and social support of experimental group compared to the control group.

Also, the results of current research indicate significant enhancement of average score of knowledge about testicle and testicular self-examination in experimental groups 3 and 6 months after the intervention, while there observed no significant changes in the control group. The reason for this issue is the availability of educational content, representations, group discussions, and using the poster, pamphlets, and educational booklets. Before the educational intervention, the level of knowledge of experimental and control groups was low. Only 5% of the experimental group and 3% of the control group had performed testicular self-examination.

In the study of Asgharpour et al. [31] on 174 Turkish students by using the health belief model, before the educational intervention, 66.2% of students did not know TSE. In the study of Avci et al. [25] on 425 students, 56.2% of subjects had heard about TSE and 18.4% of them had known about TSE. In a study by Kuzgunbay et al. [33] on 799 students, 11.1% of studied subjects were aware of TSE and only 1% performed TSE every month. In studies of Ramin et al. [21] and Lechner et al. [20], the knowledge of studied subjects about TSE was low, however, in the study of Casey et al. [34], the general knowledge about TSE was high. In a study of Ugboma and Aburoma, 88.6% of subjects had not heard about TSE and 63% of them had not learned about TSE. In studies of Akar et al. [32], Steadman and Guine et al. [35], and McCullagh et al. [36], the educational intervention caused the enhancement of the subject’s knowledge about TSE.

The results of this research indicate the enhancement of perceived susceptibility and severity (perceived threat) in the experimental group 3 and 6 months after the intervention, however, there seemed no significant changes in the control group. It means that, after the intervention, most of the subjects of the experimental group believed that they are in exposure to TC, and by not doing self-examination and timely diagnosis, they will be infected by TC and its side effects. By increasing the self-knowledge of people about side-effects and expanses of TC treatment, their protective behaviors will be enhanced. Using educational images and films and PowerPoint and the speeches of a 58 years old man suffering from TC caused the increase of perceived threat of experimental group. In a study by Karayurt et al. [37], the educational intervention caused the enhancement of perceived susceptibility, however, the perceived severity did not change. In a study of Asgharpour et al. [31], after the educational intervention, there seemed no significant changes in perceived susceptibility and severity of studied subjects. The results of other studies are in good agreement with the results of this investigation [38–41].

The results of this research indicate the enhancement average score of perceived benefits and the reduction of an average score of perceived barriers in the experimental group 3 and 6 months after the educational intervention, while there seemed no significant changes in the control group. Presenting necessary educations about the benefits of TSE and its importance for the experimental group by asking and answering questions and group discussions caused positive changes in the experimental group. The educational intervention has a great effect on the removal of barriers for performing TSE. Vadaparampil et al. [30] showed that high perceived benefits and low perceived barriers are related to the performance of TSE. In a study by Brewer et al. [42], perceived benefits and perceived susceptibility predicted the intention of subjects for performing TSE. Asgharpour et al. [31] revealed that educational intervention causes the increase of perceived benefits of TSE after the educational intervention. Akar et al. [32], by using the HBM model on 96 nurses aged between 20 to 37 years, indicated that educational intervention caused the reduction of perceived barriers of subjects. In other similar studies, the educational intervention caused the enhancement of perceived benefits and the reduction of perceived barriers [43–45].

The results of this research show that, after the educational intervention, the perceived motivation of the experimental group about TSE enhanced, while the control group showed no changes. To have successful TSE performance, subjects should have sufficient motivation for changing, performing, and having proper behaviors. In educational sessions for the experimental group, in addition to the presentation of contents by asking and answering questions, films, and educational images and providing helper and friends groups, a motivational message was sent to the subjects once a week. In the study of Karayurt et al. [37], the educational intervention caused significant differences in the motivation of nurses for doing a breast self-examination. Norman and Conner [46] revealed that internal motivation is related to the enhancement of performance. In the study of Jeihooni et al. [47], the educational intervention based on the health belief model caused the enhancement of subjects’ motivation about preventive behaviors from osteoarthritis.

The results of this research showed that, before the educational intervention, the average score of self-efficacy of experimental and control groups was low, however, after the intervention, a significant enhancement was observed in the experimental group, while the control group had no changes. Self-efficacy is an individual’s confidence about his/her ability for performing an especial action which is related to the control of that person on the environment and his/her behaviors. People with higher self-efficacy have bigger aims and their behaviors are more pleasant [48]. In studies of Lechner et al. [20] and Guteman et al. [49], self-efficacy predicts the intention of the subject for performing TSE. In the study of Avci et al. [25], the self-efficacy of students who performed TSE was higher than students who never did TSE. In a study of Roy et al. [50] on 150 Irish men aged between18 to 45 years, the knowledge of subjects about TSE was low and there was a relationship between self-efficacy and TSE performance. In the study of Akar et al. [32], the educational intervention based on HBM caused significant enhancement of subjects’ self-efficacy.

Cues to action is a factor related to the perceived social pressures and internal motivations (internal and external cues to action) which leads subjects to take TSE behavior [13]. In the present research, cues to action are the initial symptoms of TC, family members, specialists, health center personals, and friends in which their influence as an information source for performing TSE is important. In this research, one session was held with the presence of a family member, specialist and health center personals, and friends group. The obtained results indicate significant enhancement in an average score of cues to action in the experimental group after the educational intervention. In the study of Kuzgunbay et al. [33], subjects obtained their information through the internet, social media, and then from school and friends. In studies of Koshti-Richman et al. [51] and Dachs et al. [52], the role of nurses and specialists in the promotion of TSE was mentioned. In a study by Vadaparampil et al. [30], the recommendation of doctors was related to the regular TSE performance of subjects. The results of other studies are in good agreement with this study [53, 54].

In this research, 3 and 6 months after the intervention, the average score of social support in the experimental group, compared to the control group, enhanced. In studies of Lechner et al. [20], Wynd et al. [28], Brubaker and Wickersham [55], Weist and Friman [56], Barling et al. [57], and Tuinman et al. [58], there was a significant relationship between social support and TSE. In a study by Rorito et al. [59] on students aged beteen18 to 35 years, the subjective norms predicted TSE. In the study of Trumbo et al. [60], the subjective norms affected the intention for doing TSE.

In a study by Ord-Lawson and Fiteh [61], there was no significant relationship between social support and the mood of TC in patients. The results of other studies showed that the educational intervention caused the enhancement of the average score of social support in studied subjects [62–64]. In the present research, before the educational intervention, there seemed no significant differences in the average score of TSE behaviors in experimental and control groups. However, 3 and 6 months after the intervention, the average score of self-examination behavior of the experimental group enhanced more significantly than the control group, indicating the positive effect of educational intervention based on health belief model and social supports on TSE performance of studied subjects.

In the study of Mc Clenahan et al. [13] on 195 students aged between 18 to 39 years, the health belief model determined 56% intention and 21% TSE behavior. In the study of Vadaparampil et al. [30], 46% of subjects performed TSE regularly and 51% reported that they do not perform TSE regularly. In the study of Wynd et al. [28], 64% of studied subjects never performed TSE and 36% performed TSE in few months. In studies of Cox et al. [65], Mc Cullagh et al. [36], and Akar et al. [32], the educational intervention caused the enhancement of TSE performance. In a study by Akca et al. [66], the rate of TSE among the students was previously reported as 19.6% and increased to 100% after the training.

The major limitation of the present study is its self-reporting and consideration 3 and 6 months after the intervention follow-up phases to evaluate the effectiveness of educational intervention in the present study. Therefore, it is recommended to investigate the subject of the present study in multiple centers, and longer follow-up time points after intervention can also be helpful.

## Conclusion

The obtained results revealed that education based on health belief model and social support has led to this issue that, by increasing the average score of construct, the experimental group had better TSE performance than the control group. According to the sensibility and vulnerability of men aged between 15 to 35 years and the importance of earlier diagnosis of TC and the important role of social supports such as family members, specialists, and health centers personals, the demand for presenting fundamental solutions and appropriate educational programs for TSE becomes more important. In this investigation, the important role of social groups for taking TSE behaviors was mentioned and a combination of health belief model and social support construct was employed.

Being community-based research due to the selection of people who were under the coverage of health centers is one of the advantages of the present investigation. One limitation of this study was self-reporting answers of subjects about TSE performance.

## Data Availability

The datasets used and/or analyzed during the current study can be made available by the corresponding author on reasonable request.

## Abbreviations

HBM: Health Belief Model
TC: Testicular cancer (TC)
TSE: Testicular Self-Examination (TSE)
